# International Travel Health Risks and Post-Repatriation Care in Japan

**DOI:** 10.31662/jmaj.2025-0468

**Published:** 2025-11-28

**Authors:** Chihaya Hinohara, Soichiro Saeki

**Affiliations:** 1 International Health Care Center, National Center for Global Health and Medicine, Japan Institute for Health Security, Tokyo, Japan; 2 Department of Emergency Medicine and Critical Care, National Center for Global Health and Medicine, Japan Institute for Health Security, Tokyo, Japan; 3 Division of Public Health, Department of Social Medicine, Graduate School of Medicine, The University of Osaka, Osaka, Japan

**Keywords:** travel medicine, international medical transfer, insurance policy, universal health coverage, hospital management

We read with great interest the recent review by Tsukada et al. ^(1)^ on the health risks faced by international business travelers (IBTs) and the urgent need for tailored health management strategies. Their article highlights not only the wide range of infectious, psychological, and environmental risks encountered during travel, but also systemic gaps, such as insufficient pre-travel consultation and limited corporate health support.

While this review emphasizes risks during the travel period, our recent study suggests that challenges continue after travelers return to Japan. At our national tertiary hospital (National Center for Global Health and Medicine, Tokyo, Japan), we retrospectively analyzed medical repatriation cases over a three-year period (further details on the study available in Supplementary Materials). Among all inquiries, approximately one-third resulted in admission (Figure 1a), and most cases were from Asia (Figure 1b). Notably, nearly one-quarter of admitted patients were hospitalized, not for acute treatment, but for entry coordination (Figure 1c), and one-third were for patients residing out of Tokyo Prefecture (Figure 1d), reflecting the absence of immediate access to rehabilitation or long-term care facilities. Such admissions highlight a structural misalignment, whereby acute care hospitals are compelled to provide transitional entry points for patients whose conditions are already stable.

**Figure 1a.**
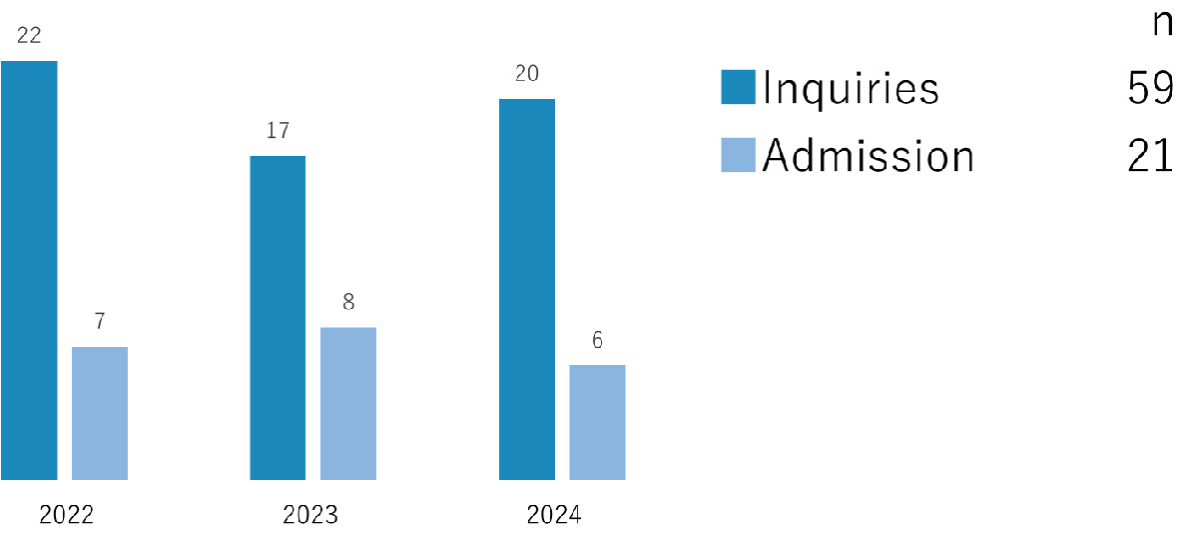
Number of inquiries and admissions per fiscal year.

**Figure 1b.**
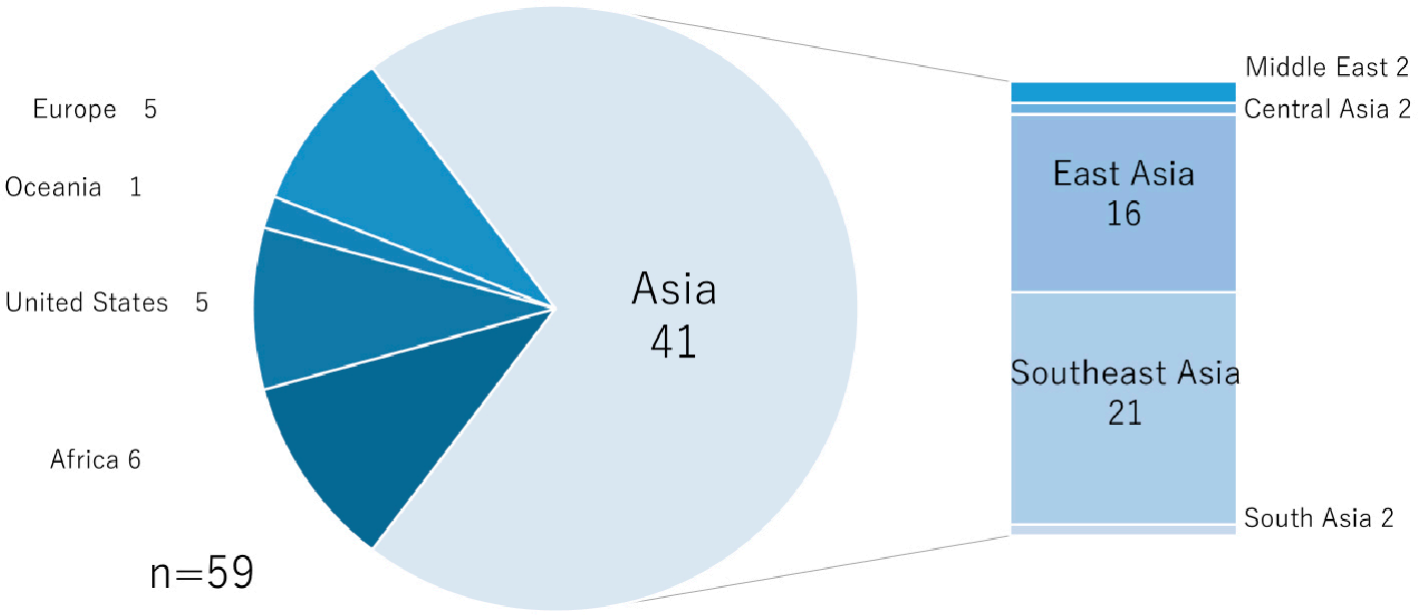
Overview of the area of patients.

**Figure 1c.**
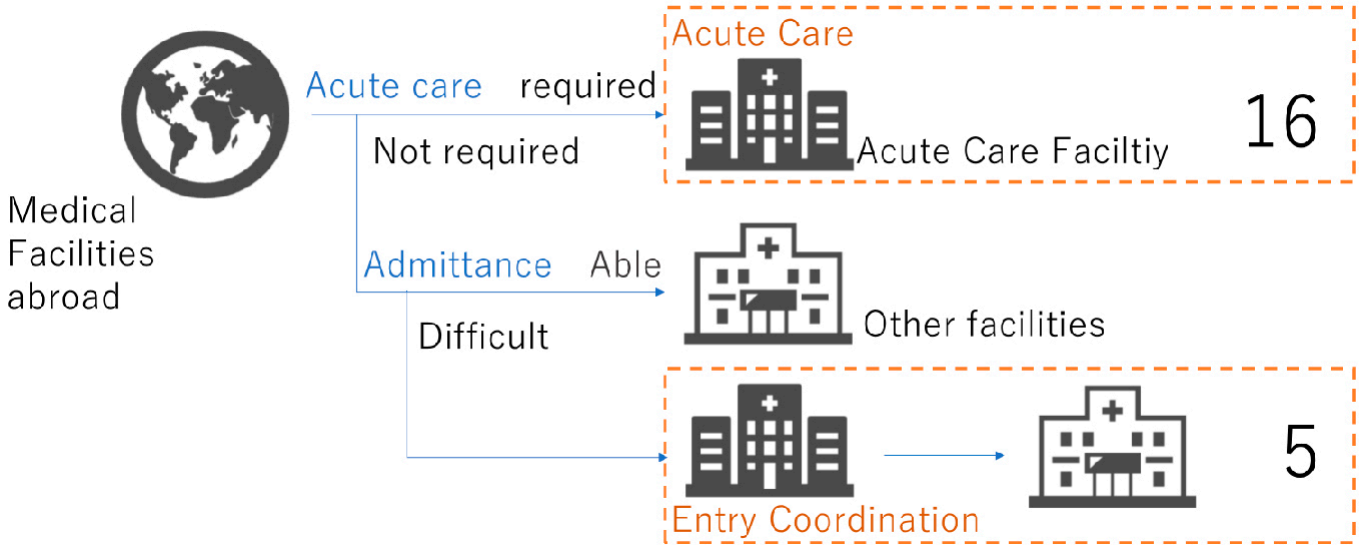
Overview of accepted cases.

**Figure 1d.**
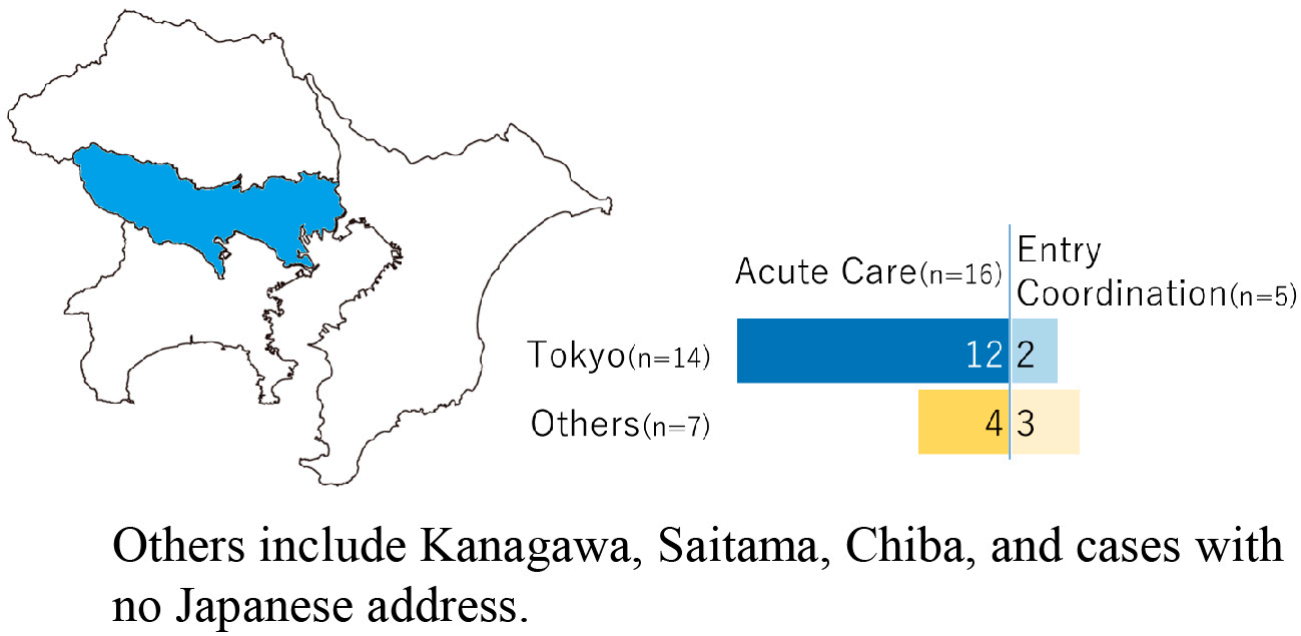
Residential address in Japan of the accepted cases.

Another important finding was the high prevalence of multidrug-resistant organisms (MDROs) detected in more than half of admitted patients (Figure 1e). While extended-spectrum beta-lactamase producers had little impact on care transitions, vancomycin-resistant enterococci posed substantial barriers, leading to prolonged hospital stays due to difficulty in arranging transfers. Recognizing this risk, the Disease Control and Prevention Center of the Japan Institute for Health Security has issued guidance documents on infection control for medical institutions, including recommendations for the management of MDROs in repatriated patients ^(2)^. Nevertheless, the practical burden on frontline hospitals remains considerable.

**Figure 1e.**
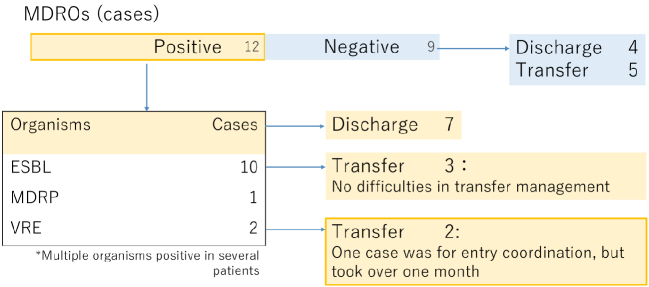
Results of multidrug-resistant organisms (MDROs) screening of accepted cases.

Although our study was not solely focused on IBTs, taken together, our results extend the implications of Tsukada et al. ^(1)^. The health risks of Japanese travelers do not end upon return; instead, they continue into the domestic healthcare system, where gaps in transitional care infrastructure and MDRO management complicate reintegration. This “second stage” of risk is under-recognized within current occupational health frameworks and deserves greater policy attention.

We advocate for an integrated strategy that bridges pre-travel and post-repatriation care. Potential measures include designating regional repatriation centers, establishing standardized pathways for non-acute cases, and strengthening national coordination for MDRO-positive patients. Such efforts would reduce the strain on acute care hospitals and ensure safer, more efficient continuity of care for Japanese citizens returning from abroad.

## Article Information

### Acknowledgments

We would like to express our gratitude to the patients who participated in this study. The authors thank their colleagues for helpful discussions on this topic, especially the Department of Emergency Medicine and Critical Care, International Healthcare Center (ICC) of the National Center for Global Health and Medicine. The authors acknowledges the use of Paperpal (Cactus Communications Services Pte. Ltd., Singapore) and Grammarly (Grammarly Inc., San Fransico, USA) for primary language editing. The views expressed in this manuscript are those of the authors and do not necessarily represent the authors institutions. The authors institutions played no role in the conceptualization of this manuscript. The abstract of this study was presented at the 29th Annual Meeting of the Japanese Society of Travel and Health (19-20 July 2025, Nara, Japan).

### Author Contributions

Conceptualization, Chihaya Hinohara; methodology, Chihaya Hinohara; software, Chihaya Hinohara; validation, Soichiro Saeki; formal analysis, Chihaya Hinohara; investigation, Chihaya Hinohara; resources, Chihaya Hinohara; data curation, Chihaya Hinohara; writing―original draft preparation, Soichiro Saeki; writing―review and editing, Chihaya Hinohara; visualization, Chihaya Hinohara; supervision, Chihaya Hinohara; project administration, Soichiro Saeki and Chihaya Hinohara. All authors have read and agreed to the published version of the manuscript.

Artificial intelligence technology was used for the language editing process, and the author reviewed such content. The author’s institution played no role in the conceptualization of this manuscript. Both authors contributed equally to this manuscript.

### Conflicts of Interest

None

### IRB Approval Code and Name of the Institution

The study was conducted in accordance with the Declaration of Helsinki, and approved by the Certified Review Board of Japan Institute for Health Security (CRB3250001, approval number JIHS-S-005108-00, approved date August 27, 2025).

## Supplement

Supplementary Material
