# Supplementary material for: International Travel Health Risks and Post-Repatriation Care in Japan
Source: JMA J. 2025 Nov 28;9(1):426–9. doi: 10.31662/jmaj.2025-0468 (PMC12889162; doi:10.31662/jmaj.2025-0468)
Supplement: Supplementary Material [file 2433-3298-9-1-0426-s001.pdf]

Supplementary Materials: Detailed methods of the study.

Methods

Study Design and Setting

This was a retrospective descriptive study conducted at the National Center for Global Health and Medicine (NCGM), a tertiary care hospital located in Tokyo, Japan. NCGM frequently serves as a receiving facility for **medical repatriation** cases—patients who require post-travel medical care after falling ill or being injured abroad.

Study Period and Population

We included all cases for which inquiries regarding medical repatriation to NCGM were made between April 1, 2022, and March 31, 2025. Both cases that were ultimately accepted for admission and those that were not were reviewed. For admitted patients, additional clinical and logistical details were analyzed.

Data Collection and Variables

The dataset included the following variables for each case: country and region where the illness or injury occurred, patient nationality, fiscal year of inquiry, referring agency (e.g., assistance or insurance companies), and transport arrangements. Diagnoses were classified into major clinical categories, such as infectious diseases, trauma, cerebrovascular events, and malignancies. The stated purpose of admission was categorized as either continued acute-phase treatment or entry coordination, defined as temporary admission to an acute care hospital due to the unavailability of direct transfer to a subacute or long-term care facility.

For admitted patients, we collected additional variables including patient residence (Tokyo vs. outside Tokyo), arrival schedule and logistics (e.g., airport and hospital arrival times), length of hospital stay, discharge destination (home, transfer), and time required to coordinate subsequent transfers.

We also documented the results of screening for multidrug-resistant organisms (MDROs), including extended-spectrum beta-lactamase-producing organisms (ESBL), multidrug-resistant *Pseudomonas aeruginosa* (MDRP), and vancomycin-resistant enterococci (VRE). Screening was conducted upon admission, and until results were confirmed, patients were isolated in private rooms under contact precautions.
